# The Effect of Adherence to Dietary Tracking on Weight Loss: Using HLM to Model Weight Loss over Time

**DOI:** 10.1155/2017/6951495

**Published:** 2017-08-09

**Authors:** John Spencer Ingels, Ranjita Misra, Jonathan Stewart, Brandon Lucke-Wold, Samantha Shawley-Brzoska

**Affiliations:** West Virginia University, Morgantown, WV, USA

## Abstract

The role of dietary tracking on weight loss remains unexplored despite being part of multiple diabetes and weight management programs. Hence, participants of the Diabetes Prevention and Management (DPM) program (12 months, 22 sessions) tracked their food intake for the duration of the study. A scatterplot of days tracked versus total weight loss revealed a nonlinear relationship. Hence, the number of possible tracking days was divided to create the 3 groups of participants: rare trackers (<33% total days tracked), inconsistent trackers (33–66% total days tracked), and consistent trackers (>66% total days tracked). After controlling for initial body mass index, hemoglobin A_1c_, and gender, only consistent trackers had significant weight loss (−9.99 pounds), following a linear relationship with consistent loss throughout the year. In addition, the weight loss trend for the rare and inconsistent trackers followed a nonlinear path, with the holidays slowing weight loss and the onset of summer increasing weight loss. These results show the importance of frequent dietary tracking for consistent long-term weight loss success.

## 1. Introduction

Type 2 diabetes (T2DM) affected over 29 million adults across the United States in 2015 and is projected to rise to over 35 million adults by 2040 if steps are not taken to slow or reverse this trend [1]. The health burden of diabetes is extensive: it is the leading cause of adult blindness, kidney failure, and nontraumatic amputations [2]. The impact of diabetes is strongly felt in West Virginia, where, in 2014, 12% of the population were diagnosed with T2DM versus 9.1% nationally in the U.S. [3]. Economic burden is high as individuals with T2DM are estimated to have medical costs 2.3 times greater than someone without this chronic condition. This is primarily due to associated comorbidities and complications [4]. The growing prevalence of diabetes in WV has led to excessive health and economic burdens for the state that require cost-effective interventions to manage and prevent T2DM.

Several evidence-based programs show that interventions to encourage lifestyle modification are effective in reducing risk for T2DM and improving glycemic control. The U.S. Diabetes Prevention Program (DPP) is a benchmark program that aimed to assess the impact of lifestyle change to manage prediabetes [5]. The project assessed the effectiveness of the lifestyle intervention (24 weeks, 16 sessions) as compared with a medication (metformin) and placebo control group. The program effectively lowered the incidence of T2DM over the 3 years of study by 58 percent in the lifestyle intervention versus 31 percent in the metformin group. The results suggest that targeted behavior change lowered risk of T2DM and improved participants' health. Similar effectiveness of a lifestyle intervention was found in the Sydney Diabetes Prevention Program [6], the Finnish Diabetes Prevention Study [7], and the Da Qing IGT and Diabetes Study [8], with each showing the usefulness for managing or preventing T2DM.

Dietary modifications are a common feature of lifestyle interventions; participants are encouraged to track their dietary intake through food journals and logs. However, it is unclear of what is the effect of increased dietary tracking on health outcomes. Participants in lifestyle management programs have cited the importance of self-monitoring dietary intake and physical activity to their success [9], yet limited studies have examined the benefits of increased self-monitoring. Participants in a 12-week weight loss program who tracked with their preferred method (e.g., pen and paper versus web-based service) were more adherent to tracking but did not significantly differ in weight loss [10]. A second study had participants track food intake with pen and paper, a memo pad on their phone, or with calorie tracking application on their phone [11]. Those who tracked with the application tracked significantly more days, but again, no difference in weight loss was noted between groups [11]. While neither study showed that better adherence to dietary tracking led to an increase in weight loss, the small sample sizes and lack of accounting of preprogram weight were limitations. However, in a 6-month study controlling for participant demographics, preprogram weight, program attendance, and physical activity for 1685 participants found increased adherence to dietary tracking-predicted weight loss [12]. Additional positive results were noted among participants who consistently adhered to dietary tracking with self-monitoring booklets over 8 weeks [13] and 12 weeks [14]; they lost more weight than those who were inconsistent in their tracking. While participants in lifestyle management programs report the importance of tracking in relation to improved outcomes, the research to date has shown mixed results.

This study will extend the current research on the impact of adherence to dietary tracking on weight loss to a 12-month community-based Diabetes Prevention and Management (DPM) program. Participants were required to track dietary intake over the project period which provided a large number of data points (22 sessions) to examine patterns between tracking and weight loss. The aim of this study is to determine the impact dietary tracking has on weight loss success over the course of a 1-year program. No research to date has tracked weight loss over the course of the year; thus, a second aim will be to use Hierarchical Linear Modeling (HLM) [15] to control for clustering and to determine what model (e.g., linear, quadratic, and cubic) best fits the participants' weight loss progress over the course of the year.

## 2. Materials and Methods

### 2.1. DPM Program Design and Implementation

The 22-session educational program is a modified DPP that uses educational material from the DPP [5] and the American Association of Diabetes Educators 7 Self-Care Behaviors (AADE7) [16]. The DPM program was implemented at two churches in West Virginia, one in Morgantown and the other in Charleston. The educational sessions covered a variety of topics related to weight loss, exercise, nutrition, diabetes prevention, and management, as well as behavioral changes (e.g., stress management and negative thinking). The sessions were conducted with the entire group at each location weekly for the first 3 months, biweekly for the second 2 months, and monthly for the final 7 months.

The group sessions were participatory and interactive over 60 minutes, rather than didactic. Session activities fostered problem solving, group interactions and social support, skill development such as reading food labels, calculating calories and fat, and setting achievable goals for each week. Many sessions included guided physical activity, food demonstrations, or model meals. The intervention was tailored to the participants' preferences and readiness to change with careful attention to cultural appropriateness for the target populations. Each session program encouraged participants to set and develop reasonable short-term goals and behavioral action plans toward dietary modifications and moderate physical activity of 150 minutes per week. Food tracking booklets and pedometers were provided to monitor their dietary and physical activity levels.

Participants were encouraged to do the following: (1) maintain daily food journals and physical activity records; (2) reduce portion sizes; (3) reduce foods high in calories, fat, and simple sugar; (4) increase consumption of fruits, vegetables, and low-fat dairy products; and (5) weigh themselves frequently and at least weekly. To promote accountability, participants weighed in at the beginning of each session and reported their minutes of physical activity and the number of daily diet records kept each week. Tracking of dietary intake included total calories and fat grams (using a 2016 CalorieKing book [17] provided to them) as well as a physical activity using step count from a pedometer provided to the participants each week. Participants turned in their food tracking books at each educational session and received feedback and individualized encouragement to improve lifestyle behaviors; they also received new blank booklets at each session to use for the following week.

Participants were assigned with a health coach (HC) who worked one on one with them throughout the program to identify threats to their plans and goals and to develop and review coping strategies. HCs contacted their participants each week to check in on progress, answer questions, and provide support. Check-ins were conducted via phone, text message, or email depending on the preferences of the participant.

### 2.2. Participant Recruitment

Participants were recruited from the two communities through the following: advertising in print media, local email lists, sending recruitment materials to be distributed to all eligible participants visiting a Federally Qualified Health Center and free clinic, and posting flyers and brochures in public places such as community bulletin boards (physical and electronic), physician's offices, grocery stores and restaurants, churches, and word of mouth. A prescreening was conducted at a convenient location at several times over the course of a few weeks prior to the program start date, in order to review the program objectives, assess if they met the criteria for participation (i.e., have type 2 diabetes or prediabetes diagnosis), and answer questions about the requirements for participation in the program.

### 2.3. Food Tracking Booklets

The tracking was completed by pen and paper with booklets that contained columns for describing food, type and amount, and listing fat, calorie, and carbohydrate content. There was also a section for tracking exercise at the bottom of each day. HCs reviewed and provided written feedback to their participants' food journals. The feedback focused on recognizing positive changes, providing general encouragement, and discussing additional information on modifications of their current dietary habits so that they make healthier choices on what to eat and not to eat. These journals, with feedback, were returned to the participants at the subsequent session.

Participants were encouraged to refer to nutrition labels when available and were provided a copy of *The CalorieKing, Fat & Carbohydrate Counter 2016* [17] in order to look up calorie, fat, and carbohydrate contents of their food. A research assistant scanned each tracking booklet, which was used to calculate the total days tracked by week for participants throughout the program period. In order to qualify as having tracked for the day, the food journal needed to have at least one meal tracked (e.g., breakfast, lunch, or dinner). The final data contained the number of days a participant tracked of the dietary information by each week and over the course of the program.

### 2.4. Measures

Participants were weighed at each session (in a private room for confidentiality) by a trained HC. In addition, waist circumference, lipid profile (total cholesterol, LDL, HDL, and triglyceride), and HbA1c were measured at baseline, midprogram or 6 months, and 12 months. At baseline, participant's height was measured for calculating their body mass index (BMI) score. Demographic information and the participants' self-reported diabetes status (prediabetes or diabetes) were also collected.

## 3. Plan of Analysis

The data was analyzed using Hierarchical Linear Modeling (HLM) software [15]. Model building was done in three steps: (1) building the best equation to model weight change over time (e.g., linear, quadratic, and cubic), (2) building the best model with control variables, and (3) building the best model with tracking variables. The 1st aim of the study, to determine the impact of dietary tracking on weight success, was answered with the results from step 3. The 2nd aim, to determine what model best fits the participants' weight loss progress over time, will be answered with the results from step 1.

**Figure 1:**
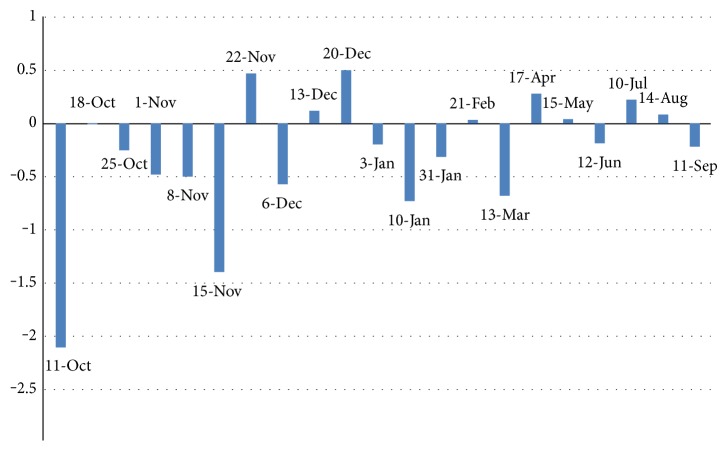
Bar chart showing average weight loss in pounds across participants at each session.

Weight loss across the 22 sessions was used as a repeated measure at level 1. Weight loss progress was graphed over the 22 sessions (49 weeks) of the program (see Figure 1), and week 1 weight loss was removed from level 1 and added as a control at level 2 in order to better model weight change over time. Time was centered at the last session of the program in order to create meaningful intercept that represented the average weight change between week 2 and week 49, after controlling for week 1 weight change, BMI, HbA1c, and gender. Centering time at the end point created a negative time variable (e.g., week 49 = 0, week 47 = −2, and week 40 = −9), so a negative intercept represented weight loss over the course of the program. In order to capture a possible nonlinear relationship in weight change over polynomial time, terms were calculated and tested (e.g., quadratic, cubic, and quartic).

**Figure 2:**
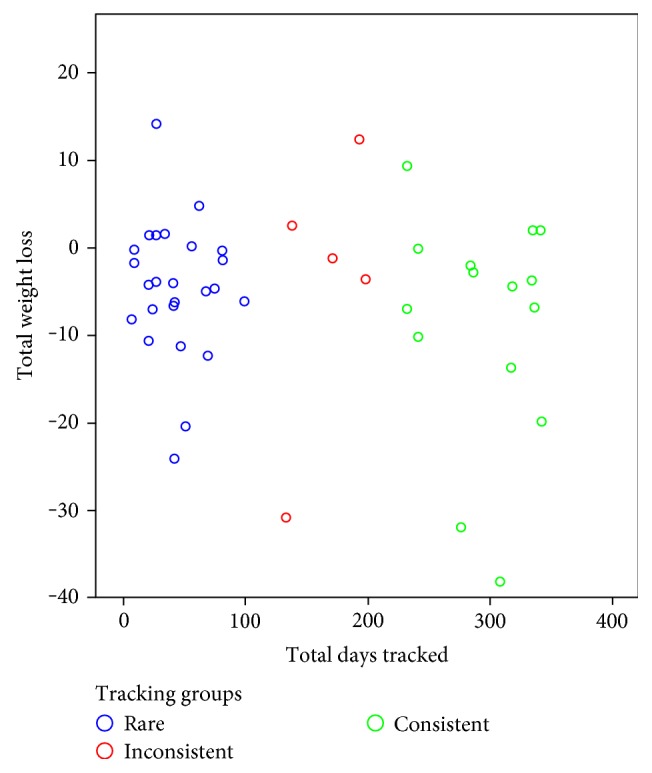
A scatterplot of total days tracked by total weight loss in pounds per participant.

Control variables included BMI, HbA1c, and gender at level 2 to determine the effect tracking had on weight over time. A scatterplot of total days tracked and total weight loss indicated a nonlinear relationship (Figure 2). To best capture this relationship between tracking and weight loss, three tracking groups were created: rare, inconsistent, and consistent trackers. Participants were split into groups with those that tracked less than 33% of total days possible (rare trackers, *n* = 25), 33–66% (inconsistent trackers, *n* = 5), or over 66% (consistent trackers, *n* = 15). Tracking groups were dummy coded twice, with rare tracking as the referent group and inconsistent and consistent tracking groups as the two focal groups. Gender was used as a control variable in the model and was effect coded as it is a categorical predictor in the regression model. BMI and HbA1c were each centered at their grand mean. Therefore, the intercept would represent the average weight loss for participants in the rare tracking group after controlling for gender, HbA1c, and BMI. The time coefficients represent the rare tracking group weight loss slope over time after controlling for gender, HbA1c, and BMI.

## 4. Results

### 4.1. Descriptive Statistics

As part of a larger study, assessing the effectiveness of lifestyle modification on health outcomes, this study included participants enrolled in 2015-2016 for the DPM program in West Virginia. Sixty-six participants completed the baseline clinical, anthropometric, and behavioral screening. However, 21 participants were excluded due to excessive missing data resulting with 45 participants (M = 13, F = 32, average age = 61.2 ± 10.7 years) in the final model. Participants attended an average of 15 (out of the 22) intervention sessions, and three-fourths attended ≥16 sessions (i.e., at least 75% of the sessions). However, participants who missed one or more session had the opportunity to view the recorded session via a closed YouTube channel created for only program participants by site. Twenty-four participants self-reported being diagnosed with diabetes, and the rest reported they had prediabetes (confirmed by HbA1c values). Average total weight loss was 5.6 pounds (SD = 12.0), average week 1 weight loss was 1.7 pounds (SD = 2.5), and average days tracked was 94.9 days (SD = 110.5). See Table 1 for a baseline descriptive data by rare, inconsistent, and consistent trackers.

### 4.2. Correlations

Bivariate Pearson's correlations showed no significant association between baseline weight, BMI, gender, HbA1c, diabetes status, total tracking days, and total weight loss. Week one weight loss (*r* = 0.37, *p* < 0.01) and consistent tracking (*r* = −0.28, *p* = 0.02) were significantly correlated with total weight loss. Since none of the anthropometric measurements were statistically significant and due to our small sample size, they were not included in the multivariate model.

### 4.3. Level 1 Model

A cubic model was the best fit for the data as compared to the quadratic model *X*^2^ (1, *N* = 2) = 14.13, *p* < 0.001. A quartic model could not be run due to overlap in the variance explained between the cubic and quartic terms. To examine the within- and between-participant variability, the intraclass correlation coefficient (ICC) was calculated. ICC showed 64% between-participant variability existed in total weight change, while 36% within-participant variability existed for weight change over time. In addition, we checked the assumption of normality in error using a histogram of the residual variance at level 1 and level 2, which showed the variance to be normally distributed.

### 4.4. Level 2 Model

A control model, using week 1 weight loss, BMI, HbA1c, and gender to predict both the intercept and the slopes at level 2, provided the best fit to the data. These variables failed to significantly predict final weight loss or weight change over time, but adding the tracking variables resulted in a statistically significant improvement in the control model *X*^2^ (8, *N* = 2) = 19.35, *p* = 0.01. The final level 2 model explained 15.31% of the variance between participants' final weight loss, as compared to the control model.

### 4.5. Predicting Final Weight Change

Consistent tracking was a statistically significant predictor of average weight change over the course of the program (*β*_06_ = −7.59, *p* = 0.04), while inconsistent tracking (*β*_05_ = 4.97, *p* = 0.34) and rare tracking (*β*_00_ = −2.40, *p* = 0.30) were not associated with statistically significant weight loss. After controlling for gender, BMI, week 1 weight loss, and HbA1c, only those participants who consistently tracked their diet lost a statistically significant amount of weight, that is, an average of 10 pounds.

### 4.6. Changes in Weight over Time

**Figure 3:**
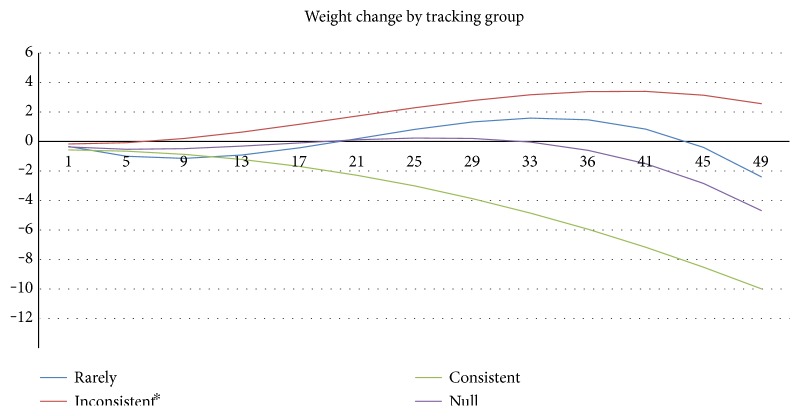
Predicted weight loss, in pounds, over time across tracking groups, from week 2 to week 49, holding all other variables constant. Inconsistent curve is not statistically different from the rare curve. Week 9 is the thanksgiving week and the beginning of the holiday season, and weight begins to climb. Week 33 is the beginning of summer (June), and we see weight start to drop again. However, consistent trackers do not experience these changes and sustain steady weight loss throughout the year. ^∗^Nonsignificant.

Figure 3 shows the pattern of weight loss over time by participants over the 49-week program period, controlling for other variables in the model. Rare and inconsistent trackers lost weight initially but did not sustain it over time. However, with the progress of time, the quadratic coefficient increased at a rate faster than that of the linear coefficient in the model suggesting that these two groups of participants will regain their initial weight loss. Not until the 33rd week or the 8th month of the program is the cubic time coefficient large enough to overcome the quadratic term's weight gain prediction. The weight gain corresponds with participants going through the holidays (Thanksgiving through the New Year), and the weight loss in the 33rd week corresponds with the beginning of summer (June). However, consistent dietary trackers could sustain weight loss over time (see Figure 3). In other words, these individuals did not experience the cyclical fluctuations and maintained a consistent weight loss over time as the cubic coefficient minimized the effect of the quadratic coefficient. Hence, consistent trackers' weight loss more closely resembles the weight loss predicted by the initial linear time coefficient of losing approximately 2/3 of a pound per week. See Table 2 for full reporting of all coefficients across the null, control, and final models.

## 5. Discussion

Tracking is an important component of success in lifestyle change programs [9], but there is limited research on the effect of dietary tracking adherence on weight loss [10–14]. The DPM program tracked participants' weight over 49 weeks, at each of the 22 sessions, which allowed the examination of the role of dietary tracking on weight loss over time. The results showed that individuals who completed over 228 days of food logs to track their dietary intake (out of a possible 343) lost a significant amount of weight, as compared with those who tracked less than 228 days. Furthermore, participants who tracked between 114 and 228 days did not lose a significant amount of weight. In terms of predicting weight loss, individuals who consistently track 5 or more days a week were successful in losing and sustaining weight loss over the course of the year. Our results concur with research by Boutelle and Kirschenbaum [13] who found that consistent trackers lost more weight in an 8-week study. Our results extend these findings by showing consistent tracking continues to be beneficial over the course of a 1-year program, highlighting the benefit of consistent tracking as a safe and sustainable effort to improve long-term weight loss success.

While studies present equivocal benefit in weight outcomes with increased adherence to tracking, an 8-week study by Wharton et al. [11] found that those who tracked with mobile phone application tracked more days than those who tracked with either pen and paper or the memo pad on their phone, yet weight loss did not vary between the groups. However, those in the pen and paper and memo groups received nutritional counseling prior to the start of the program and received weekly emails to encourage healthy eating. The application group received no dietary advice outside the information from the nutritional software on total calories and macronutrient consumption. Hence, it is possible that the weekly emails or the nutritional counseling prior to the start of the program may have hidden the effect-increased adherence to dietary tracking of participants in the mobile phone application group on their final weight change. The results from the current study provide emerging evidence on the impact of increased adherence to dietary tracking on weight loss due to the standardized assessment of all participants in the program.

Another (similar) study did not find a difference in weight change among active duty overweight military members (BMI ≥ 25 kg/m^2^) who tracked with their preferred method (phone, pen and paper, or web-based application) versus their nonpreferred method [10]. Each participant was provided with the same nutritional feedback and support through group classes, similar to the DPM program. However, this 12-week study did not model weight change over time, did not control for participant characteristics, and was completed with a group of military personnel who differ from the general population, which may have minimized the ability to detect the impact of consistent tracking on weight change.

The current study found that a polynomial model provided the best fit for the participant weight data as it created a curvilinear slope to best fit weight change over time. As expected, we detected greater weight loss for the majority of the participants during the first quarter of the program, as seen by the large effect of the linear time term, with this effect lessening over time. However, the quadratic time coefficient decreased the rate of weight loss, while the cubic time coefficient increased the rate of weight loss. Finally, the nonlinear trend of weight change was logical since the model with session dates revealed that the slowing in weight loss corresponded with the beginning of the holidays; similarly, the increase in weight loss in the last quarter of the program was associated with the beginning of summer months. This finding concurs with a qualitative research study that reported participants face more barriers to healthy behaviors during the holidays [18]. However, with the beginning of summer, weight loss becomes easier as the warm weather allows participants to do outdoor activities, exercise, and shop for healthier food options.

Our results suggest that consistent tracking had a significant impact on weight change over time. Tracking over 228 days did not have an effect on the linear, or instantaneous, rate of change. Early in the program weight change of consistent trackers did not differ from rare or inconsistent dietary trackers. However, rare or inconsistent trackers gained weight during the holidays but the consistent trackers' rate of weight loss did not change as they sustained their rate of weight loss from the first quarter. Hence, consistent dietary tracking seems to have a protective effect on participants' holiday eating challenges as they were able to survive the holiday parties and managed their holiday stress without adding extra pounds to their weight. Perhaps, consistent tracking helped these participants stay on track with programmatic goals and topics, such as planning healthy snacks and meals during the holiday season and being aware of fat, sugar, and calorie consumption. Thus, the rate of weight loss for consistent trackers closely followed the initial rate of change shown at the beginning of the program, as consistent tracking cancelled out the effect of the quadratic and cubic terms. This indicates that consistent tracking predicted more stable weight loss over time. However, future research should explore other factors that might impact weight loss to better understand both the process by which consistent tracking impacts weight loss as well as the outcome of sustainable weight loss. It is possible that consistent tracking allowed participants to be more mindful of their dietary habits and be motivated to avoid high calorie, fat, and sugary items (e.g., eating a cookie) during the holiday season to avoid writing it down in their tracking book. Additionally, the participants who consistently track may be more compliant and thus more likely to track and follow dietary recommendations of the program. Hence, it is possible that participant's personal characteristics could impact tracking adherence and weight outcomes.

Consistent tracking can also be viewed as a measure of resilience or ability to stay on track during challenging times (like the holiday season). As shown in the graph (Figure 3), the weight loss over time for a participant who consistently track followed a stable, negative linear trend. In contrast, weight loss over time for those who rarely or inconsistently track was unstable, as they lost weight in the first and last quarter of the program, while they gained weight during the middle, during the holiday seasons (Thanksgiving and Christmas), and during the winter months. The weight loss predicted during the second half of the program, that is, spring and summer months, may be explained by an increased motivation to change dietary habits in order to fit into beachwear and summer clothes for increased outdoor activities as the temperature warms up during this time of the year in West Virginia.

It is interesting to note that the rare trackers (i.e., had food journals for less than 114 days) and the inconsistent trackers (i.e., those who had food journals and tracked their dietary intake between 114 to 228 days of the year) did not have significant or sustainable weight loss over the program period for the 12 months. Hence, successful behavioral interventions should emphasize the benefits of consistent dietary tracking for participants, motivating individuals to track for at least 5 days of each week for sustained and clinically significant weight loss. Inconsistent tracking of dietary intake did not impact weight loss, and individuals did not differ in their overall weight loss from those who rarely track. This data concurs with what has been reported in prior research [12, 13].

## 6. Limitations

A primary limitation was that the DPM program was a pre-post, uncontrolled intervention study, with the majority of participants who are non-Hispanic Whites (which are representative of the state). Furthermore, a quarter or 26% of participants had missing final weight information due to either missing the postintervention assessment or dropping out and were therefore excluded from the analysis. Another limitation was the use of self-reported adherence to diet. Hence, the model represents those who completed the program and the dietary tracking and not all program participants. The low number of participants who inconsistently track may have limited the ability of the model to detect a statistically significant effect over time. Treating tracking as a level 2 variable alone does not provide a complete evaluation of the relationship between tracking and weight loss over time; it instead treats tracking as a static variable that does not vary weekly and over the program period. Hence, future research can enter number of days tracked as a time varying covariant in order to determine the effect of tracking on weight change throughout the program. While dietary tracking shows participants' level of commitment to tracking, weight loss is influenced by factors other than diet (such as physical activity, level of stress, medication management, and comorbidities) that need to be included in future models. Finally, more research is needed on larger samples to confirm this finding and to expand our understanding of the relationship between tracking and weight change over time.

## 7. Conclusions

In summary, dietary tracking was found to be an important component of successful weight loss, with those who tracked at least 5 days of each week showing significant and sustained weight loss over time as compared to those who tracked fewer days or inconsistently during the program. Consistent tracking is a significant predictor of weight loss, resulting in an additional seven pounds of weight loss over the course of the program suggesting the intervention successfully achieved clinically and significant long-term weight loss in high-risk rural Appalachian adults with diabetes and prediabetes. In addition, a model of weight change over time revealed that more weight was lost over the summer as compared with the holiday season. Despite potential challenges to eating healthy during holidays, those who consistently maintained their food journal and tracked their calories and fat intake did not experience an increase in weight over the holidays, indicating that consistent tracking may act as a protective factor to the challenges of following a healthy lifestyle during the holidays. Future research can test this hypothesis by looking at changes in dietary tracking over time to determine if certain periods of tracking are critical to weight loss success.

## Figures and Tables

**Table 1:** Descriptive statistics across the 3 tracking groups: rare (<33% of days tracked, *n* = 25), inconsistent (33–66%, *n* = 5), and consistent (>66%, *n* = 15).

	Rare	Inconsistent	Consistent
Gender	M = 7, F = 18	M = 1, F = 4	M = 5, F = 10
College graduate	17	2	3
Income		Missing = 1	Missing = 1
<25k	4	1	3
25–50k	7	0	9
50–75k	5	1	2
75–100k	6	0	0
>100k	3	2	0
Mean days tracked (s.d.)	43.2 (25.2)	166.6 (30.2)	294.9 (42.0)
Mean baseline weight in pounds (s.d.)	225.8 (44.6)	205.5 (71.9)	202.3 (52.6)
Mean weight loss in pounds (s.d.)	−4.6 (7.7)	−4.1 (16.1)	−8.5 (12.9)
Mean baseline HbA1c (s.d.)	6.97 (1.32)	6.86 (2.22)	6.65 (1.21)
Mean baseline BMI (s.d.)	36.4 (6.3)	34.9 (9.4)	34.2 (7.6)

M = males; F = females; s.d. = standard deviation.

**Table 2:** Comparison of model parameters (standard error) across the null, control, and final models on final weight outcome in pounds.

	Null model	Control model	Final model
*Intercept*			
Intercept2	−4.69 (1.75)^a^	−4.58 (1.88)^b^	−2.40 (2.29)
Wk-1 loss		−0.37 (0.65)	0.03 (0.62)
BMI_0		−0.39 (0.25)	−0.45 (0.23)
HbA1c_0		1.00 (1.28)	0.49 (1.20)
Gender		0.33 (3.83)	1.49 (3.56)
Inconsistent			4.97 (5.15)
Consistent			−7.59 (3.60)^b^
*Linear time slope*			
Intercept2	−0.53 (0.07)^a^	−0.46 (0.08)^a^	−0.61 (0.12)^a^
Wk-1 loss		0.01 (0.03)	0.003 (0.03)
BMI_0		−0.01 (0.01)	−0.01 (0.01)
HbA1c_0		−0.01 (0.05)	−0.002 (0.05)
Gender		0.30 (0.16)	0.28 (0.23)
Inconsistent			0.42 (0.23)
Consistent			0.22 (0.16)
*Quadratic time slope*			
Intercept2	−0.02 (0.003)^a^	−0.02 (0.004)^a^	−0.03 (0.01)^a^
Wk-1 loss		0.0004 (0.001)	−0.0007 (0.001)
BMI_0		0.0002 (0.0005)	0.0004 (0.0005)
HbA1c_0		−0.004 (0.002)	−0.003 (0.002)
Gender		0.007 (0.007)	0.004 (0.008)
Inconsistent			0.016 (0.01)
Consistent			0.024 (0.007)^a^
*Cubic time slope*			
Intercept2	−0.0002 (0.00005)^a^	−0.0002 (0.00005)^a^	−0.0003 (0.00007)^a^
Wk-1 loss		−0.0000 (0.00002)	−0.00002 (0.00002)
BMI_0		0.00001 (0.00001)	0.00001 (0.00001)
HbA1c_0		−0.00006 (0.00003)	−0.00005 (0.00003)
Gender		0.00002 (0.0001)	−0.00002 (0.0001)
Inconsistent			0.0002 (0.0002)
Consistent			0.0003 (0.0001)^a^

^a^ *p* < 0.01, ^b^*p* < 0.05, (standard error).
